# A Bungling Chemist’s Discovery

**Published:** 1890-01

**Authors:** 


### A Bungling Chemist's Discovery.

In the course of conversation at Cornell University, Edward Atkinson, the Boston economist, stated that a New England genius has recently discovered a cheap method of dissolving zinc by combining it with hydrogen, and producing a solution called zinc water. This liquid, if applied to certain woods, notably white wood, makes it absolutely fire-proof, and at a low cost. Mr. Atkinson regards this discovery as one of the most important of the age, and one that will surely revolutionize fire insurance, as well as immensely decrease the loss by fire. The invention is kept secret for the present. Only one foreigner—Sir Lyon Playfair, the English scientist—knows of it. He corroborates all that is claimed for the invention, and says that the inventor is a bungling chemist, but that he has a faculty of blundering into the choisest secrets of nature's laboratory. As soon as patents are perfected and capital interested, zinc water will become an article of commerce.—*Sanitary Volunteer.*

